# Improving wetland management through First Nations’ knowledge and a spatial visualisation tool

**DOI:** 10.1007/s13280-025-02326-2

**Published:** 2026-02-23

**Authors:** Maria Fernanda Adame, Emad Kavehei, Bex Dunn, Sue Jackson, Phil Duncan, Minjerribah-Moorgumpin Elders in Council, Jacqueline Cahill, Natasha Nadji, Christopher James Brown, Leo Lymburner

**Affiliations:** 1 Australian Rivers Institute, Griffith University, Nathan, QLD 4111 Australia; 2 Geosciences Australia, Australia Government, Canberra, ACT 2609 Australia; 3 University of Canberra Galambany Professorial Fellow, Centre of Applied Water Science, Canberra, ACT 2617 Australia; 4 Minjerribah-Moorgumpin Aboriginal Elders in Council, Dunwich, QLD 4183 Australia; 5 University of Tasmania, Churchill Ave, Hobart, TAS 7005 Australia

**Keywords:** Cultural values, Macrophyte invasion, Monitoring, Water extraction, Waterholes

## Abstract

As wetlands continue to decline, there is an urgent need to improve their management by following the guidelines of international programs. The Ramsar Convention on Wetlands requires assessing baseline conditions and monitoring, for which Earth Observations are powerful tools. However, spatial and temporal limitations require integrated co-management, incorporating First Nations' Knowledge. We address three case studies with unique management challenges in Australia: invasive macrophytes in tropical Kakadu (Bininj/Mungguy Country), water extraction in subtropical Minjerribah-Terrangeri (Nunukul, Goenpul and Ngugi Country), and agriculture in semiarid Gwydir wetlands (Kamilaroi/Gamilaroi/Gomeroi/Gamilaraay Country). We utilise spatial imagery visualised with the Wetlands Insight Tool (WIT), which detects long-term changes (1988–2021), along with UAV imagery and First Nations’ Knowledge, to understand the baseline condition of the wetlands and the changes they are experiencing. This approach can support decision-making on the threats to address, the solutions to take, and the monitoring of the world’s remaining wetlands.

**Supplementary Information:**

The online version contains supplementary material available at 10.1007/s13280-025-02326-2.

## Introduction

Freshwater wetlands are one of the most valuable ecosystems for human survival in the Anthropocene. They provide clean water and food, as well as intangible values such as spiritual connection to the land, forming an integral part of many cultures. The ecosystem services provided by wetlands, which support human well-being, are becoming increasingly important in achieving the 2030 Sustainable Development Goals, specifically improving water quality (Target 6.3), promoting sustainable food production (Target 2.4), and ensuring the adequate management of natural resources (Target 12.2; Jaramillo et al. 2019). Wetlands are highly threatened, with 35% of their area (potentially up to 87%, Davidson 2014) lost in the past 50 years (Bridgewater et al. 2021). Losses have been attributed primarily to land-use changes caused by agriculture, water extraction, pollution, and the introduction of invasive species (Bridgewater et al. 2021). Moreover, climate change is affecting wetlands through increases in temperature and changes in rainfall patterns (Bunn 2016).

As wetlands continue to decline, there is an urgent need to improve their management in line with the guidelines of international programs and policies. For instance, the Ramsar Convention on Wetlands requires assessing wetland baseline conditions and continuing monitoring in a format useful for managers and other end-users (Mackay et al. 2009). However, monitoring has been inadequate, and changes in the ecological character of wetlands (Article 3.2) are reported by fewer than 20% of the Ramsar Contracting Parties (Davidson et al. 2020). Long-term monitoring and analyses of the data are essential to understand the condition and apply adequate and adaptive management (Hansen et al. 2021). For these purposes, Earth Observations are promising tools (Mackay et al. 2009); however, they have limitations in resolution at small scales (< 10 ha) and are typically unavailable before the 1970s or in cloudy conditions, which are common in the tropics. Finally, interpreting and visualising spatial imagery can be complex and require specialised skills.

A more comprehensive approach to Earth observations involves working in partnership with First Nations peoples, whose knowledge is deeply embedded in places through a holistic approach of long-term and continued environmental observations (Berkes and Berkes 2009). First Nations communities often possess fine-grained, detailed information about local ecosystem patterns and processes, which can be valuable for natural resource assessments, especially in areas where extant systems of customary resource management prevail and Western scientific knowledge is limited or non-existent. This way of learning has already proven successful in various wetlands worldwide. For instance, in Honduras, the Traditional Knowledge of indigenous fishermen, combined with hydrological analyses and modelling, has provided solutions to mitigate the effects of dam construction on the fish populations of the Patuca River (Esselman and Opperman 2010). In Northern Australia, Traditional and Western Knowledge have been combined to assess the long-term impacts of feral pigs on the condition of waterholes (Russell et al. 2021). This combined approach aligns well with international initiatives such as the Intergovernmental Platform on Biodiversity and Ecosystem Services, which aims to bridge First Nations and modern Western knowledge systems in proposing solutions to environmental challenges (Rathwell et al. 2015).

Despite the potential benefits, incorporating First Nations' Knowledge and translating this ‘way of knowing’ into objective measurements, which are usually required by international conventions, is challenging (Tengö et al. 2014), and implementation into practical management actions has been slow (Huntington 2000; Bridgewater and Kim 2021). For instance, legislation and national policies in Australia recognise the need for Indigenous participation in water management; however, their inclusion remains poor (Jackson et al. 2017). In this study, we conducted fieldwork in three regions in Australia, co-designing the project with the Traditional Custodians, with whom we investigated three management issues of concern to them: invasive macrophyte species in tropical Kakadu National Park, water extraction and droughts in subtropical Minjerribah-Terrangeri (Stradbroke Island), and agriculture impacts and climate change in Gwydir wetlands.

## Materials and methods

### Principles of engagement

We applied the principles of collaboration outlined in the Minjerribah-Moorgumpin Elders in Council protocol, based on reciprocity and respect, mutual benefit, and centred on learning, sharing, and contributing in a two-way manner (MMEIC 2024). We considered it essential to have all of First Nations’ partners included as co-authors of this publication. For Kakadu National Park, we also followed the engagement protocols established by the permitting processes of the Australian National Parks agency (Permits PA2020-00062 and RK.948). These include requirements to access field sites only with Traditional Owners; to pay for their time and knowledge, and to consult with them on any publications resulting from fieldwork.

We developed an early partnership with our First Nations co-authors, creating the opportunity for them to co-design the project. The Traditional Owners selected the sites and the research question that were to be investigated according to their management requirements, ensuring that the data obtained from each of the study cases would be closely aligned with their values and needs. After the field trip, the lead author travelled back to visit each of the partners and showed them the results in person, discussing the implications of the data. For our partners in Queensland, the WIT tool is available online (WetlandMaps, Queensland Government), and the lead author showed them how to use this tool independently.

### Study sites

#### Kakadu national park, Northern Territory, Australia

**Fig. 1:**
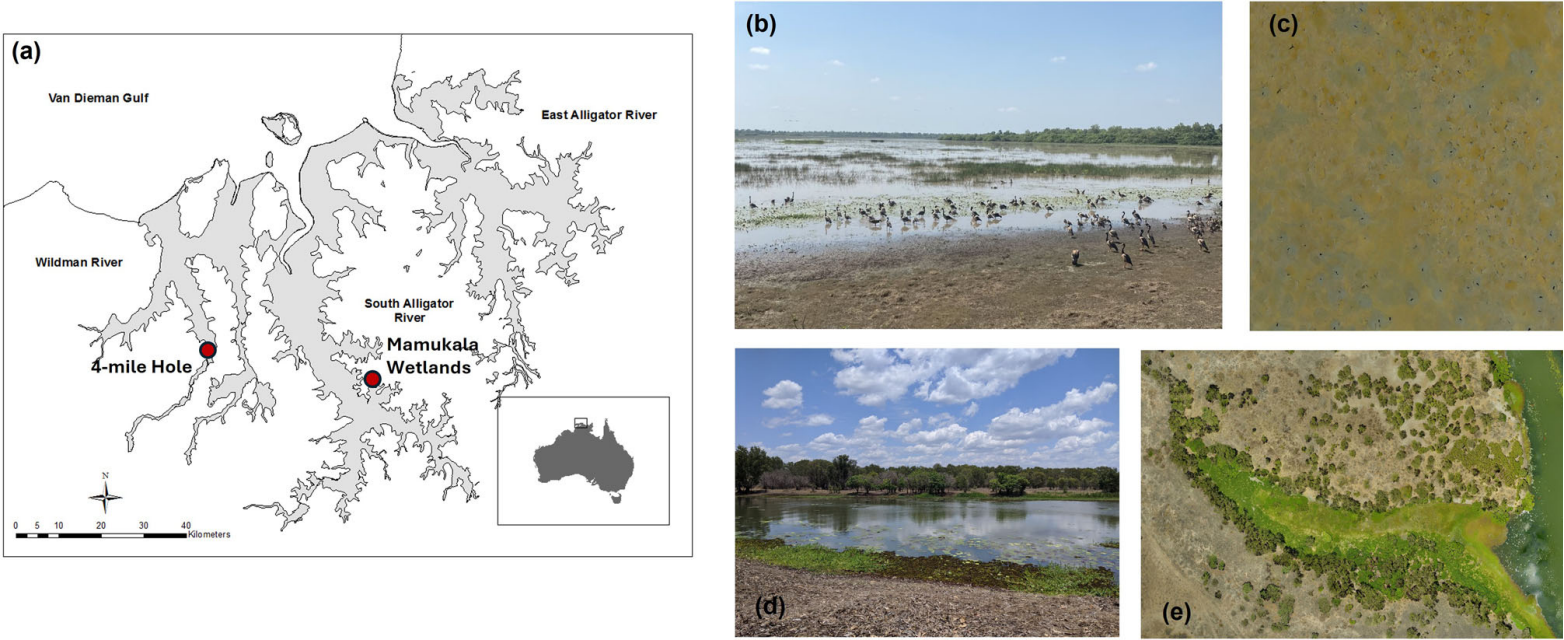
(**a**) Study sites within Kakadu National Park, Northern Australia, (**b**) Mamukala wetlands is a culturally important waterhole where large flocks of magpie geese, a valuable food source, gather during the dry season, (**c**) magpie gees feeding on the bulbs of “wild rice” (*Oryza meridionalis*), every dot is an individual goose; (**d**) 4-mile Hole is a culturally significant site where people socialise and fish, (**e**) it has been affected by the invasion of *Salvinia molesta*. Photo credit M.F. Adame

The World Heritage-listed Kakadu National Park (KNP) is in the Northern Territory and is the traditional country of the Bininj/Mungguy people. They are organised into 19 clans or family groups and have deep connections to the wetlands from which they obtain abundant food sources, or “bush tucker” (Adams et al. 2018). The KNP has a tropical monsoonal climate, with a total mean annual rainfall of 1547 mm, mostly falling between December and March (1383 mm), and an annual mean temperature range of 22.7–34.4 °C (1971–2021; Bureau of Meteorology, BM, Jabiru Airport, Station 14 198). Rainfall and water flows in KNP are strongly associated with the El Niño-Southern Oscillation and the Pacific Decadal Oscillation (Denniston et al. 2015). Two main rivers flow through KNP: the South Alligator River and the East Alligator River (Fig. 1a), which overflow and inundate their floodplains during wet periods (Ward et al. 2016). During the dry season, flooding recedes, and permanent water holes remain in the floodplain until the next wet season, when they are reconnected to the river channel. The waterholes left after the rivers retreat are critical refuges for many species of fish (Crook et al. 2020) and birds, such as “black-necked storks” (*Ephippiorhynchus asiaticus*; Dorfman et al. 2001) and “magpie geese” (*Anseranas semipalmata*; Traill and Brook 2011).

#### Minjerribah-Terrangeri, Southeast Queensland, Australia

Moreton Bay lies within Quandamooka Country and encompasses Minjerribah-Terrangeri and Moorgumpin (Stradbroke and Moreton Island), which are sand islands of high wetland diversity (Arthington et al. 2019, Fig. 2). Minjerribah-Moorgumpin is the country of the Nunukul, Ngugi, and Goenpul people, who have inhabited the islands for over 25 000 years (Fischer et al. 2019). Minjerribah-Terrangeri has a complex groundwater system that receives and accumulates rainwater, which is filtered through sand, and into freshwater palustrine and estuarine wetlands before ultimately discharging into the ocean. The climate is subtropical, with a mean annual precipitation of 1537 mm, with the highest values between January and June during the wet-hot season (1035 mm; BM, Dunwich Station, 40 537, 1960–2021). Temperature ranges between a monthly maximum of 29.6 °C in January and a mean monthly minimum of 21.2 °C in July, the dry-cool season (Point Lookout Station, BM, 40 209, 1997–2019).

**Fig. 2:**
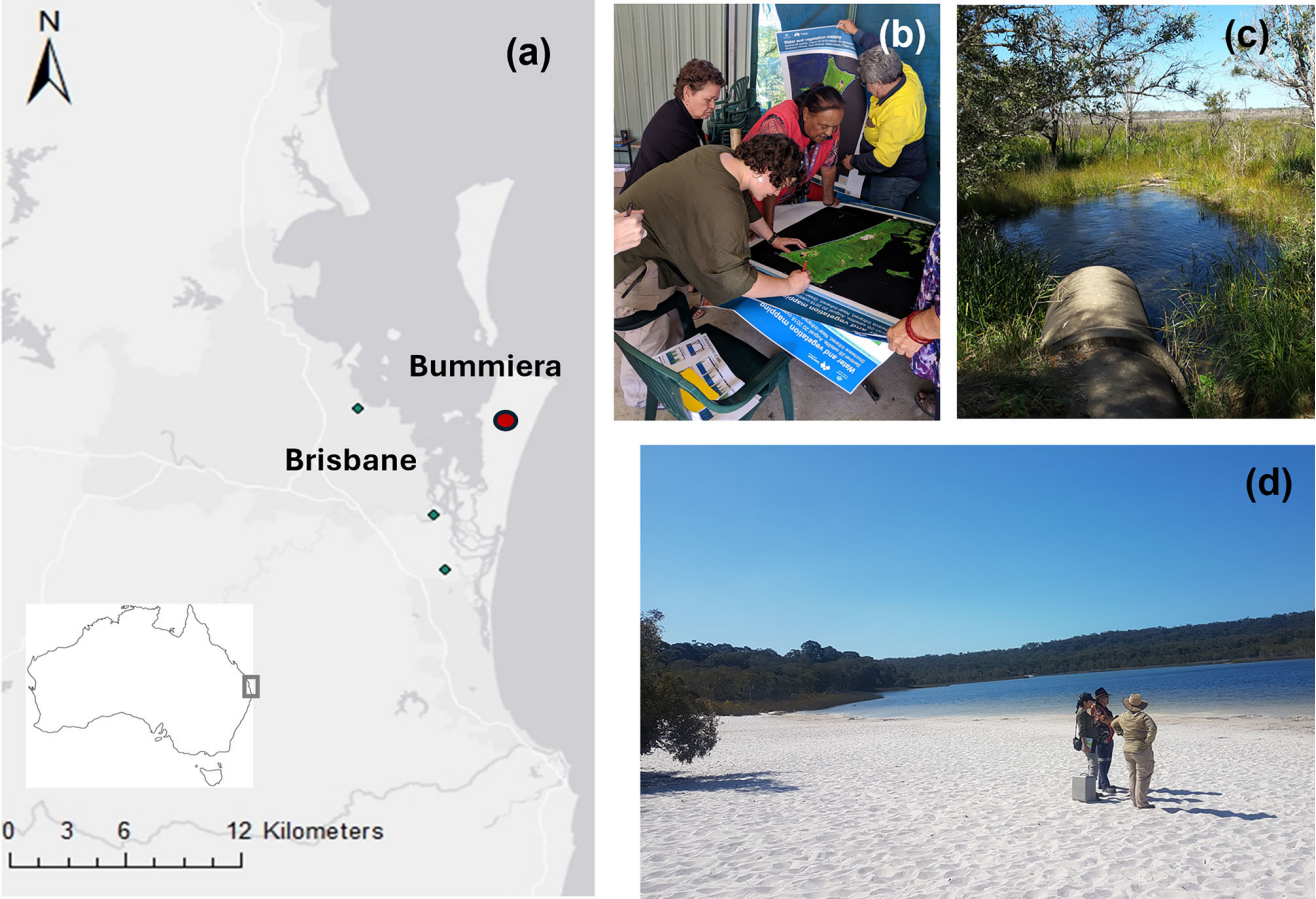
(**a**) Minjerribah-Terrangeri, in southeast Queensland, (**b**) Bummiera (Brown Lake) is a culturally significant site for the First Nations of Minjerribah. (**c**) There is concern about water extraction from the island to supply the growing demand for water on the mainland (**d**) During 2019, a very dry year, the edge of Bummiera lake was over 50 m further than its original perimeter. Picture with permission from the Elders. Photo credit M.F. Adame

#### Gwydir wetlands, Murray-Darling Basin, Australia

The Gwydir wetlands are located within the Murray-Darling Basin, which has the country’s most extensive river system, spanning a catchment of over 100 million ha, covering 14% of mainland Australia (Murray-Darling Basin Authority, MDBA 2021, Fig. 3). The Murray-Darling Basin has over 30 000 wetlands, 16 of which are internationally recognised for their importance to migratory birds (MDBA 2021). The Basin is home to over 40 First Nations groups who have cared for the land and waterways for more than 45 000 years, with a deep cultural, spiritual, and environmental connection to the land and water. Within the Murray-Darling Basin, the Gwydir wetlands are the traditional lands of the Kamilaroi/Gamilaroi/ Gomeroi/Gamilaraay peoples.

**Fig. 3:**
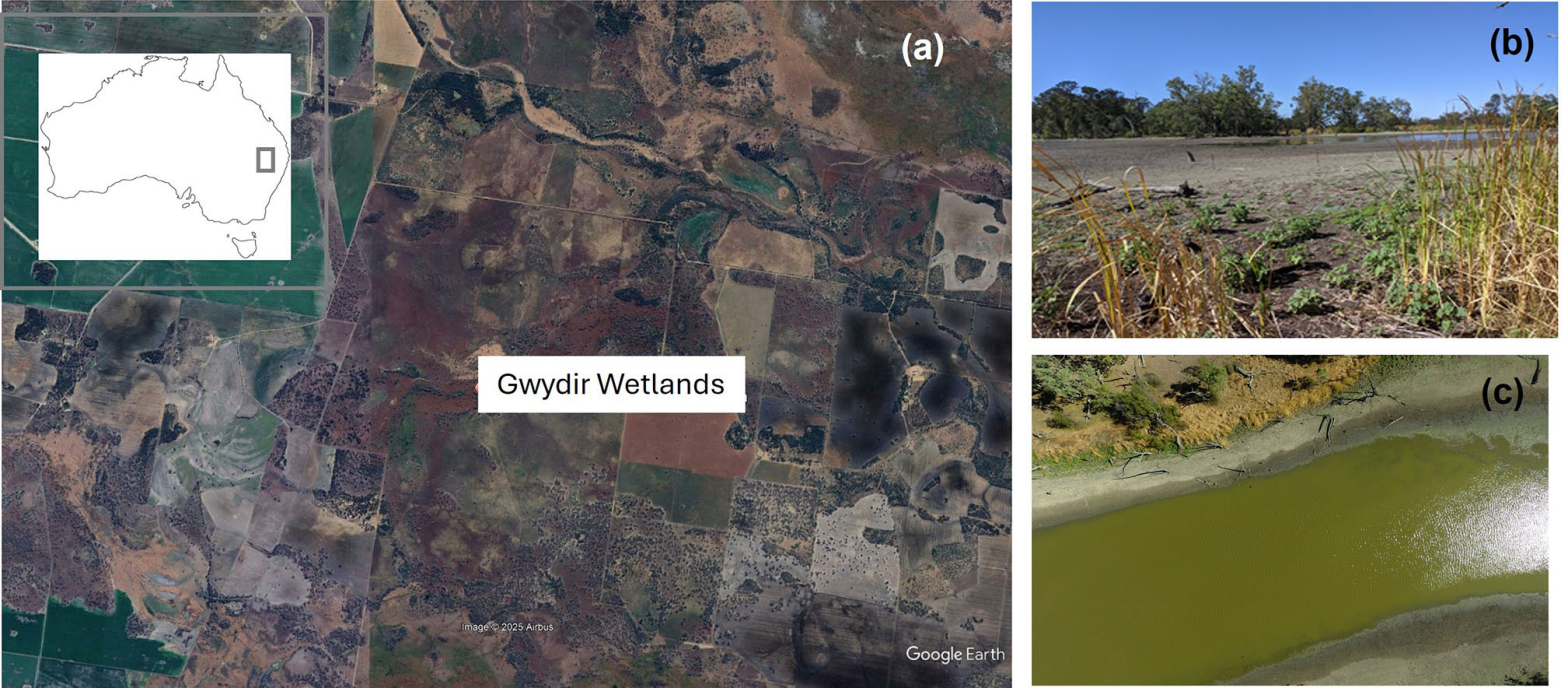
(**a**) Gwydir Wetlands are in the Murray-Darling Basin, in semiarid region with extensive agricultural land use, (**b**, **c**) Gingham waterhole in Gwydir Wetlands at the end of the dry season in December 2019. Picture from Google Earth. Photo credit M.F. Adame

### Field work

We travelled to each of the selected sites with our First Nations partners between 2019 and 2020. On-site, we discussed the changes that had been observed during the past century. We flew a UAV (Phantom 4, DJI) and created a map of the location, discussing the changes observed from the air. The main challenges for each of the identified wetlands, as summarised from direct observations and the scientific literature, are outlined in the following sections.

#### Invasive macrophytes and climate change in Kakadu National Park

A pressing issue for the wetlands of KNP is the invasion of non-native macrophytes (Adams et al. 2018). In the nineteenth century, the British colonisers introduced *Hymenachne amplexicaulis* and “paragrass” *Urochloa mutica* for pastures. The latter invades areas of “wild rice” (*Oryza meridionalis*), which is an important food source and breeding ground for the magpie geese (Adams et al. 2015; Boyden et al. 2018; Fig. 1b, c)*.* In 1980, another non-native invasive macrophyte, “Salvinia” (*Salvinia molesta*), was accidentally introduced in KNP. This invasive macrophyte has been controlled with some success by native aquatic weevils (*Cyrtobagous salviniae*), which can rapidly consume this plant (Schooler et al. 2011). The management of Salvinia has been conducted through the Djurrubu weevil breeding program, in collaboration with KNP, Northern Territory Government, and the Gundjeihmi Aboriginal Corporation. However, in years with low rainfall, low water flows have favoured its rapid expansion in ponded water, and the biological control has been less efficient.

Mamukala was selected as the site due to its cultural and ecological significance. It is a permanent water hole that is important for magpie geese, a valued food source. The practice of harvesting and managing these goose populations is an essential connection to the ancestral land (Bayliss and Ligtermoet 2018). Other waterholes, such as the 4-mile hole, are important sites for social gatherings, fishing, and hunting, and was selected as the second study site (Fig. 1).

#### Water extraction and rainfall variability in Minjerribah-Terrangeri

Minjerribah-Terrangeri is part of the Redlands Council, next to Brisbane City (Fig. 2), which has a population of 2.3 million. The population within the region is expected to grow by > 800 000 within the next two decades, undoubtedly increasing water demand (Arthington et al. 2019). SeqWater, a statutory authority of the State Government, currently manages water storage, transport, and treatment. Seqwater obtains 54% of its water for the Redlands Council from Minjerribah-Terrangeri, amounting to 8250 ML annually (SeqWater 2017). The water from the island is of very high quality due to the natural filtering capacity of the sand island and its healthy wetlands, thus requiring minimal treatment.

The Elders of Minjerribah-Moorgumpin are concerned about the effects of water extraction on the wetlands, especially those of cultural importance, such as Bummiera or Brown Lake, which they observe to be decreasing in size (Fig. 2). Lake Bummiera is one of eastern Australia’s few unique dunes or perched lakes.

#### Agriculture and climate extremes in Gwydir wetlands

After Europeans arrived in the early nineteenth century, the land surrounding Gwydir wetlands was heavily farmed, especially for cotton, wheat, cattle, and pecans (Fig. 3). Water is scarce in the region due to its semiarid climate, characterised by annual precipitation of 625 mm (1988–2020, BM, Moree, 53 033). The lack of water has degraded many wetlands, especially during droughts, which cause salinisation, cyanobacteria blooms, and fish deaths (Pittock and Finlayson 2011). The risk will only worsen with climate change, further intensifying drought conditions (CSIRO 2008). In response, a federal water law was introduced, and a multi-governmental agreement was established to provide water for the environment. The Murray-Darling Basin Authority (MDBA) was established to balance the use of water between the people who utilise it and the environment, through the Living Murray program. The goal is to improve the health of the Murray-Darling Basin through "environmental water", which is the allocation of river flows for environmental purposes.

The site selected for this project was Gwydir, due to its spiritual and cultural significance (Fig. 3). The water from these wetlands is primarily controlled by the Copeton Dam, which discharges water into the Gwydir River and the Gingham Water Course. Within the Gwydir wetlands, Gingham is a waterhole characterised by flashy hydrology, featuring short periods of flooding followed by prolonged dry periods. Following the Millennial Drought (1997–2009), the Gwydir wetlands were extremely dry, and environmental flows have been allocated since 2014 (Table 1).

**Table 1:** Environmental water flows to Gingham Watercourse during 2014–2019 (Commonwealth Environmental Water Office 2019)

Year	Commonwealth Environmental Water (ML)	NSW supplementary water (ML)	Annual total flow (ML)	Environmental Water (% total flow)
2014	15 000	14 868	46 711	64
2015	675	2375	29 043	11
2016	4259	13 741	102 667	18
2017	2000	5534	20 894	36
2018	20 000	15 000	40 443	87

### Data analyses

After the field trip, The Wetland Insights Tool (WIT) was run for each site. The WIT (Dunn et al. 2023) is a workflow that generates plots showing changes in open water, areas of water mixed with vegetation (here, called ‘wet soil’), green vegetation, dry vegetation, and bare soil surface. The WIT uses data from Digital Earth Australia (DEA) Landsat Earth Observations (Li et al. 2012), DEA Water Observations (Mueller et al. 2016), DEA Fractional Cover and Tasseled Cap Wetness (Fisher et al. 2016). The tool summarises 35 years of observations at a resolution of 30 m within a wetland boundary as a time-series plot (WIT plot; Dunn et al. 2023) and is publicly available at Australia’s open-source DEA Open Data Cube, a platform where data can be accessed, managed, and analysed (Lewis et al. 2017).

After the data were analysed, a follow-up meeting was arranged in person to review the results of the WIT and discuss the alignment or mismatch between long-term observations and the graphs produced (Table S1). Values of solar radiation, temperature (mean monthly maximum and highest, Point Lookout Station 40209; 1997–2021; mean monthly maximum and minimum, and monthly minimum and maximum, Moree Aero Station 053115; 1995–2020), and precipitation (monthly cumulative Jabiru Airport Station, 14198; 1988–2021; Point Lookout Station 40209; 1997–2021; Moree Aero Station, 053115 1995–2020) were obtained from the Bureau of Meteorology (BM 2021).

Finally, to determine trends of open water and vegetation cover, we fitted generalised additive models with smoothers for year and month (Wood 2017). The year smooth was a cubic-regression spline, the month smooth was a cyclic cubic spline. For Minjerribah, we tried models with both Gaussian and scaled *t* distributions and found the scaled-*t* gave a better fit (on visual checks of residuals), due to some outlier values, we proceeded using the scaled *t*. We present trends in the cover as variables ± standard errors.

## Results and discussion

### Invasive macrophytes and climate change in Kakadu National Park

**Fig. 4:**
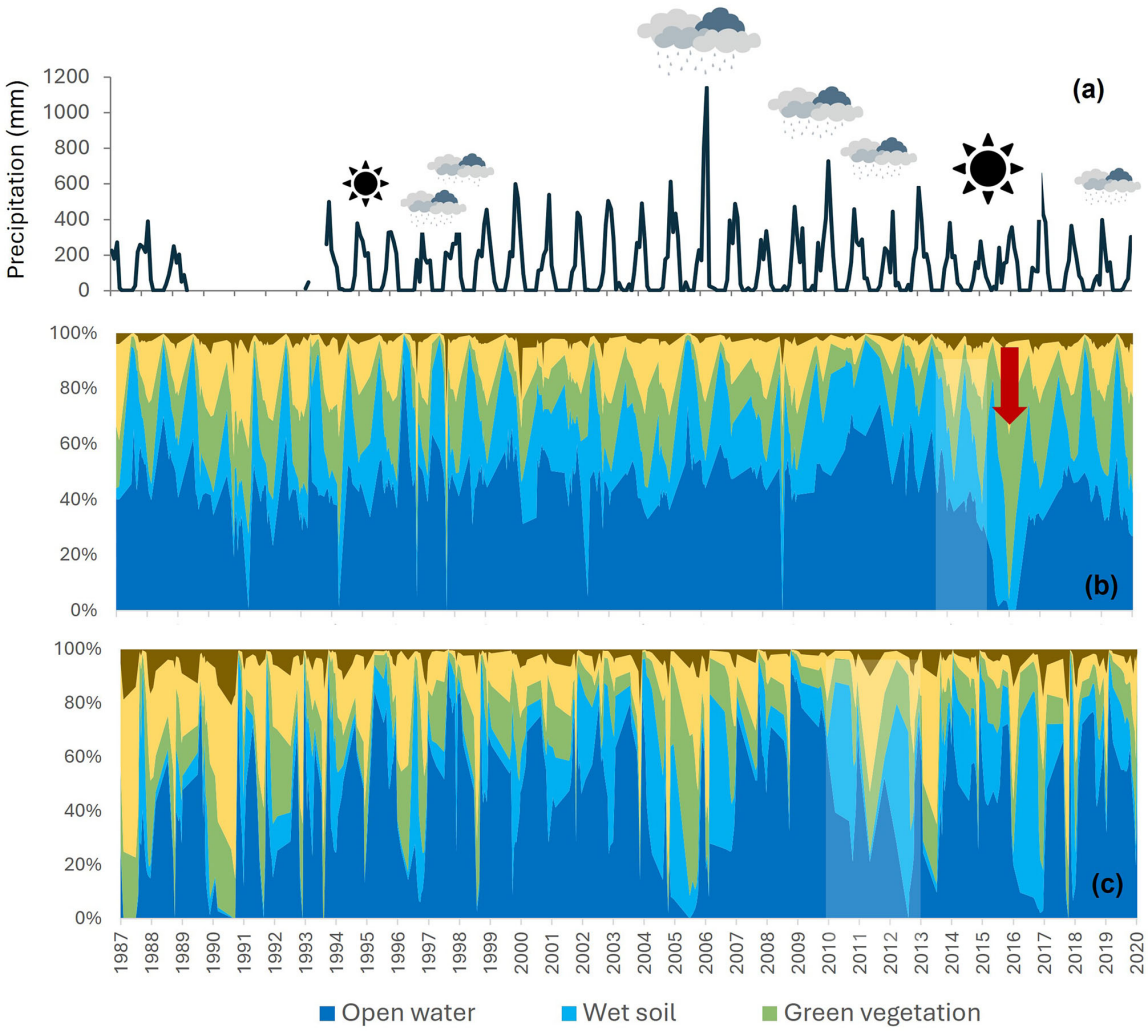
**a** Precipitation (mm), driven mainly by ENSO effects, with strong (small sun symbol) and very strong (large symbol) La Niña events causing low precipitation, and moderate (small rain symbol) and strong (large symbol) El Niño events causing high precipitation. **b** Changes in the fractional cover in 4-mile Hole and **c** Mamukala wetlands. In 4-mile Hole, an increase in green vegetation in 2016 (red arrow) reflects an unprecedented infestation of *Salvinia molesta.* Shaded areas (2011–2015) show data with uncertainties due to poor spatial images during those years

The local observation of our First Nations partner aligned well with the WIT results (Fig. 4), corroborating the occurrence of Salvinia associated with fluctuations in precipitation and temperature. During 2016 (red arrow in Fig. 4b), there was an unprecedentedly severe expansion of Salvinia, which can be attributed to the previous intense El Niño event in 2015, resulting in very low precipitation. The invasion was managed by closing the site to tourists to avoid weed dispersal and releasing weevils to control its growth. Since 2017, the extension of Salvinia has been lower, but it remains prevalent in some parts of the waterhole, as observed on-site (Fig. 1d, e).

In the Mamukala wetlands, our First Nation partner has not observed significant changes over the past few decades (GAM explains 33.8% of deviance, with a statistically insignificant change in open water from 1990 to 2020 of 0.37 ± 0.03 to 0.40 ±0.04). Aligned with the WIT results, changes in area and vegetation cover have been small and primarily driven by oscillations in precipitation between dry and wet periods, regulated by ENSO effects (Fig. 4).

### Water extraction and rainfall variability in Minjerribah-Terrangeri

**Fig. 5:**
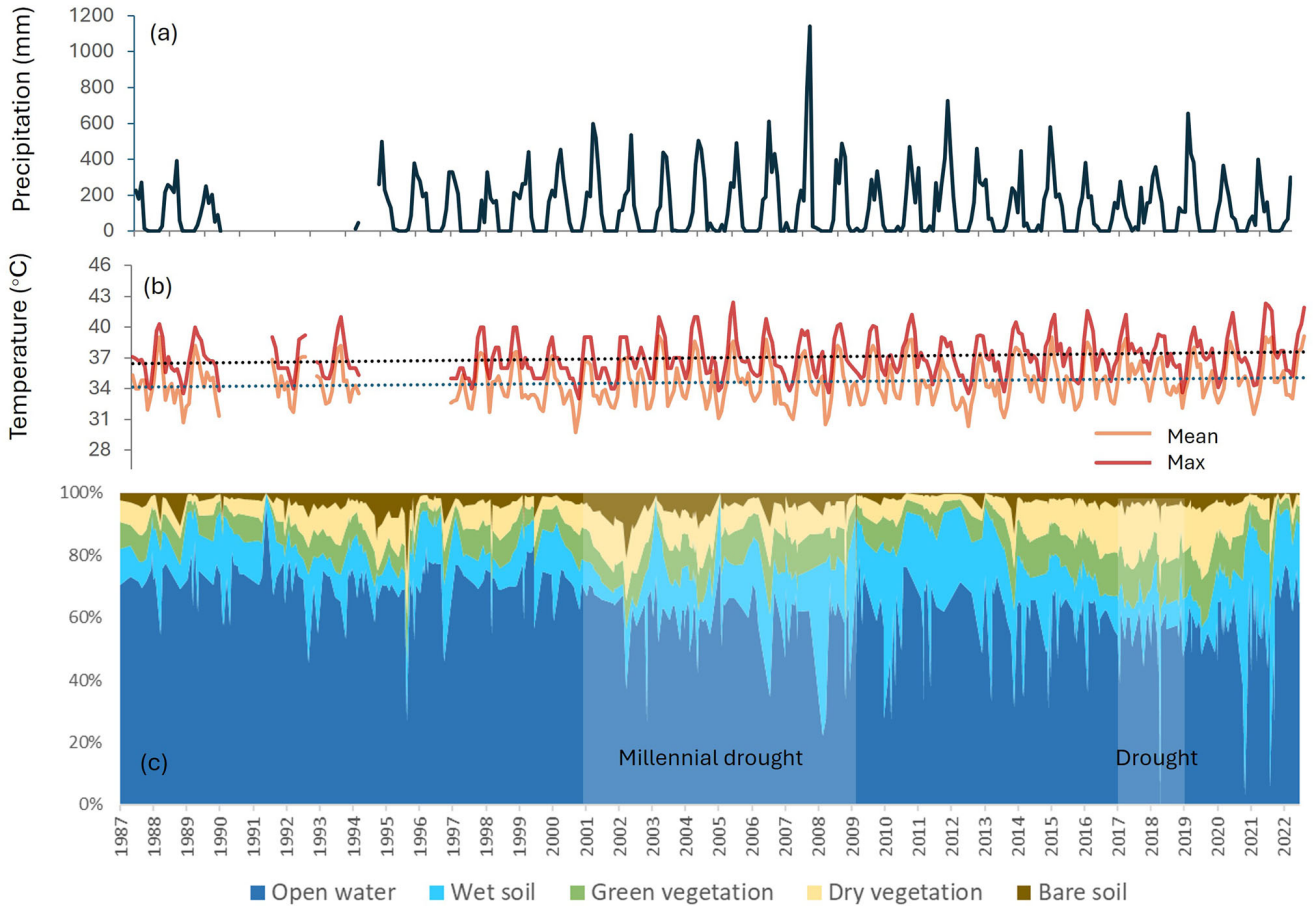
**a** Precipitation (mm) changes from 1987 to 2022, **b** significant (*p* < 0.001) increase in mean monthly maximum and highest temperatures, **c** decrease in open water area in Bummiera Lake, Minjerribah since the beginning of the Millennial Drought (2001–2009)

The observations of the Minjerribah Elders align with the WIT tool, showing that the open water area of the lake decreased by > 10% from 2000 to 2019 (model deviance explained = 35.1%, decline from 0.73 ± 0.01 to 0.62 ± 0.02 for wet-hot and 0.75 ± 0.01 to 0.63 ± 0.02 for the dry-cool season, respectively, Fig. 5). The trend in water level decrease started during a strong and long drought in Australia that occurred between 2001 and 2009 (Millenial drought; Van Dijk et al. 2013).

Monthly maximum mean temperatures are significantly increasing by 0.09 °C every summer (*R*^2^ = 0.62, *p* < 0.0001) and by 0.06 °C every winter (*R*^2^ = 0.56, *p* < 0.0001). Solar radiation has remained constant during the dry-cool season but has decreased during the wet-hot season (*R*^2^ = 0.25, *p* = 0.006). However, precipitation does not exhibit a linear increase or decrease trend (*p* = 0.59 and *p* = 0.96 for the wet-hot and dry-cool seasons, respectively). The decrease in the size of Bummiera is correlated with the increase in the mean maximum temperature, suggesting that evaporation can be partly responsible (35% of the variance explained) for the decrease in the lake’s area (scaled *t* distribution, *R*^2^ = 0.180; *n* = 282; *Z* = 238.8; *p* < 0.01).

### Agriculture and climate extremes in Gwydir wetlands

**Fig. 6:**
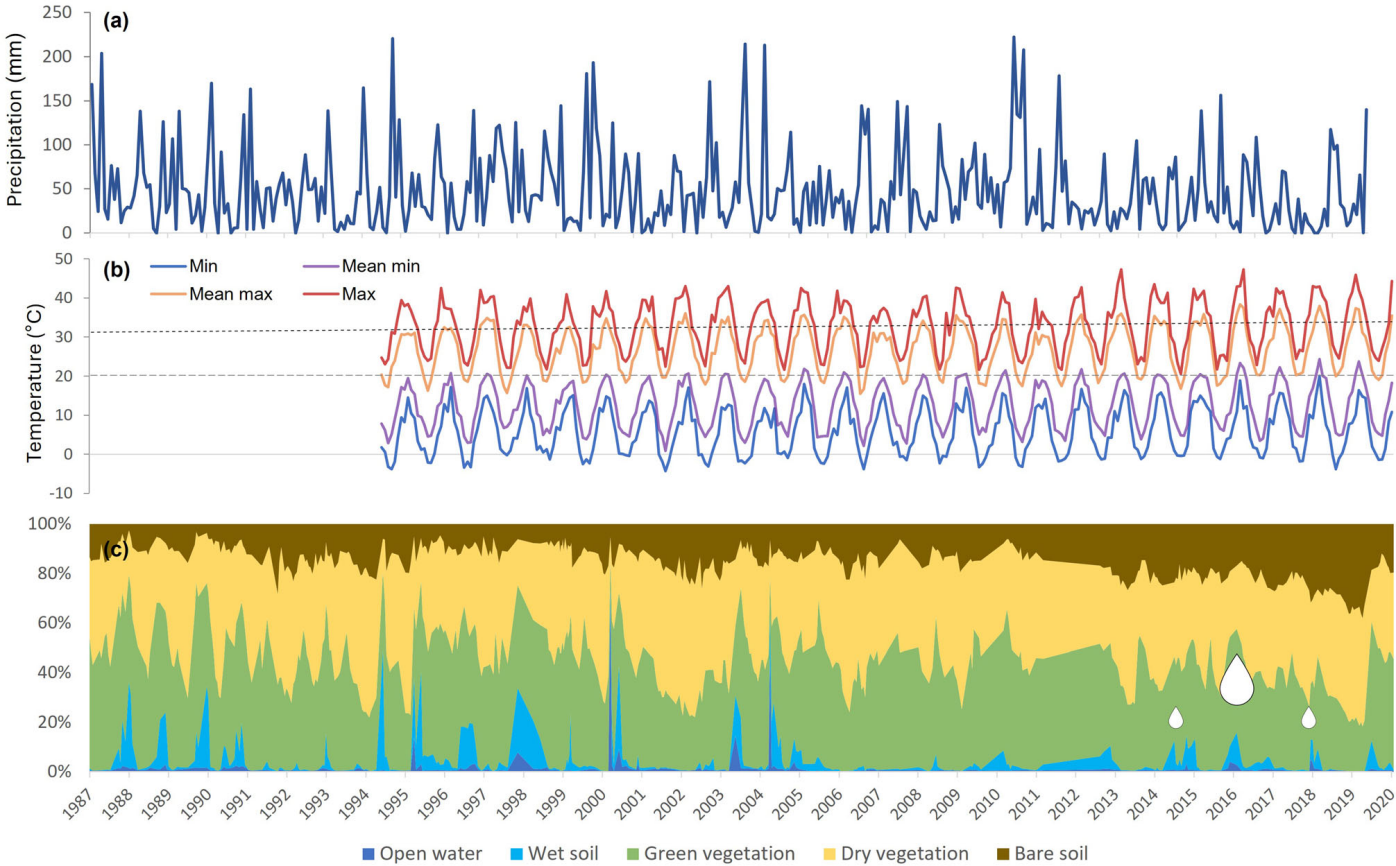
**a** Precipitation (mm) changes from 1987 to 2022, **b** minimum and maximum temperatures (°C) from 1988 to 2020, with a significant increasing trend (*p* < 0.001), **c** changes in the fractional cover in Gingham waterhole in Gwydir wetlands. Water drops represent environmental flows (see Table 1)

The observation of drying conditions in the wetland by our First Nations partner was also corroborated with the WIT tool. There was a near doubling in bare soil area from 0.13 ± 0.007 in 2000 to 0.24 ± 0.01 in 2020, but no significant change in area of open water over the same period. GAMs explained 59.5% and 2.6% of deviance for bare soil and open water, respectively. The WIT was able to identify the effects of environmental flows into the Gwydir wetlands (Table 1). These environmental flows have slightly increased the open water area, but are still small compared to years before the drought. The low water levels at Gingham Waterhole, which have persisted since the beginning of the twenty-first century, have likely been exacerbated by climate change, which has caused a significant increase in temperature in the region (*p* < 0.01, Fig. 6).

## Learnings and management recommendations

Like much of the world, wetlands in Australia face unprecedented challenges due to climate change and the continuous degradation caused by anthropogenic activities. Invasive species, temperature increases, variability in rainfall patterns, and water extraction all reduce wetland health, affecting the relationship between people and the environment. As the challenges persist, weaving First Nations and modern Western Knowledge systems could help improve the success of management and restoration actions (Mistry and Berardi 2016, 2019; Jackson et al. 2012).

In Kakadu National Park, changes in precipitation are altering inundation patterns and the habitat of magpie geese (Bayliss and Ligtermoet 2018). The WIT can be used to monitor changes in the extent of open water in Mamukala, particularly as maximum high temperatures continue to increase. This information could help determine whether suitable habitats for goose populations are available and provide guidance on sustainable harvesting sites for this culturally important species. Sea-level rise is another concern for the local community near Mamukala. The WIT, although cannot measure changes in salinity, can indicate changes in vegetation or inundation frequency that could indicate rising sea levels. Management actions to mitigate the effects of sea level rise, such as culling invasive ungulate animals that dig in the soil, facilitating saltwater intrusion (Sloane et al. 2019), could be implemented in response. The WIT was particularly useful at detecting weed invasions in the 4-mile Hole in Kakadu National Park. It provided our partners with helpful information, indicating that large Salvinia blooms are likely to be preceded by dry years, such as those during El Niño years. Introducing weevils early during dry years could be a management action that could reduce the intensity of subsequent Salvinia blooms.

In Minjerribah, the continuous monitoring of Bummiera and all the island's wetlands can be assessed online using the WIT tool, which informs communities about the potential effects of water extraction on the island. As evaporation is expected to continue increasing with high temperatures, this assessment is particularly relevant. The Traditional Custodians are currently working with the WIT to inform the future Water Management Plans of the island, which are revised every five years.

Finally, in Gwydir wetlands, the WIT was helpful to our partners for detecting whether environmental water was reaching the wetlands and for how long. This information could be used to advocate for future water allocations for these wetlands to improve their condition. The WIT could also support future “cultural flows”, which are proposed to be water entitlements owned and managed by First Nations people to improve their spiritual, cultural, environmental, social, and economic conditions (MDBA 2021). Cultural flows benefit important activities like fishing, hunting, ceremonies, and harvesting medicinal plants, and will help maintain connections to Country, cultural identity, capacity building, and intergenerational teaching (MDBA 2021). Monitoring the health and water needs of the wetlands will be essential for maintaining the cultural practices of the Kamilaroi/Gamilaroi/Gomeroi/Gamilaraay people.

Overall, we have demonstrated that the weaving of First Nations and modern Western scientific systems can support a multiple-evidence-based approach, enabling the reciprocal exchange of knowledge (Tengö et al. 2014). In this two-way knowledge transfer format (Huntington 2000; Lewis and Sheppard 2006), we provide much-needed practical examples of collaboration between First Nations and modern Western science (Rathwell et al. 2015). Globally, the potential of incorporating Traditional Knowledge into various management challenges has been demonstrated, as seen in the case of fisheries for Pacific Salmon in Canada (Atlas et al. 2021) and abalone in Mexico (Saenz-Arroyo and Revollo-Fernandez 2016). Based on our experience, the combined approach of partnerships, joint visits to field sites, and the results of the WIT tool was beneficial for all partners in understanding, learning, and proposing agreed-upon solutions to environmental challenges.

## Conclusion

In this study, we have shown how First Nations Knowledge can be woven into modern Western science to determine threats and reach agreements on the threats and solutions to the management of wetlands. The visual graphs, along with the drone images, were engaging and stimulated discussions on how wetlands have changed and how to act upon this change. The data time series information can provide a mechanism to merge multi-decadal and, in some cases, multi-generational knowledge gained through First Nations’ past and present awareness of “Country”, which is part of a living culture. Weaving spatial visualisation tools with scientific and First Nations Knowledge is a promising way of addressing the challenges that wetlands face in our times. This holistic approach can support decision-making on the threats to address, the solutions to take, and the monitoring of actions in the remaining wetlands of the world.

## Supplementary Information

Below is the link to the electronic supplementary material.

Supplementary file1 (PDF 15 KB)

## Data Availability

All the data are shown within the manuscript or publicly available (Bureau of Meteorology, Australian Government). The code for the WIT is available in Dunn et al. (2023).
