# Supplementary material for: Improving wetland management through First Nations’ knowledge and a spatial visualisation tool
Source: Ambio. 2026 Feb 23;55(8):1818–29. doi: 10.1007/s13280-025-02326-2 (PMC13319274; doi:10.1007/s13280-025-02326-2)
Supplement: Supplementary file 1 — Supplementary file1 (PDF 15 KB) [file 13280_2025_2326_MOESM1_ESM.pdf]

Supplementary Information

This Supplementary Information has not been peer reviewed

Improving wetland management through First Nations' Knowledge and a spatial visualisation tool

Adame MF, E Kavehei, S Jackson, P Duncan, N Enoch, J Cahill, N Nadji, C Brown, L Lymburner

Table S1. Questions discussed with First Nations partners on the changes observed at each wetland.

1. What are the management problems or threats that concern you most?
2. What places are you most worried about and what has been occurring there?
3. When did they become a problem, when did you notice them?
4. How can information about these problems help you and others to better manage your country?
5. What difference does it make to how you understand the problem to be able to see the changes in vegetation, soil or water (etc), over time?
6. Would you look for changes to water levels, or to soil, or to vegetation when you visit? Are these important indicators of the health of the country?
7. Are they the right indicators?
8. Do you think that these images will help others to understand what has been happening to your country?
9. Do these images miss anything that is of concern to you?
10. The images show past changes, how might they help you and other managers to predict change so that you can act now or in the future?
